# Formaldehyde impairs transepithelial sodium transport

**DOI:** 10.1038/srep35857

**Published:** 2016-10-20

**Authors:** Yong Cui, Huiming Li, Sihui Wu, Runzhen Zhao, Deyi Du, Yan Ding, Hongguang Nie, Hong-Long Ji

**Affiliations:** 1 Department of Anesthesiology, First Affiliated Hospital of China Medical University, Shenyang, Liaoning, China; 2 Institute of Metabolic Disease Research and Drug Development, China Medical University, Shenyang, Liaoning, China; 3 Department of Cellular and Molecular Biology, University of Texas Health Science Center at Tyler, Tyler, Texas, USA

## Abstract

Unsaturated oxidative formaldehyde is a noxious aldehyde in cigarette smoke that causes edematous acute lung injury. However, the mechanistic effects of formaldehyde on lung fluid transport are still poorly understood. We examined how formaldehyde regulates human epithelial sodium channels (ENaC) in H441 and expressed in Xenopus oocytes and exposed mice *in vivo*. Our results showed that formaldehyde reduced mouse transalveolar fluid clearance *in vivo*. Formaldehyde caused a dose-dependent inhibition of amiloride-sensitive short-circuit Na^+^ currents in H441 monolayers and of αβγ-ENaC channel activity in oocytes. α-ENaC protein was reduced, whereas phosphorylation of the extracellular regulated protein kinases 1 and 2 (ERK1/2) increased significantly post exposure. Moreover, both α- and γ-ENaC transcripts were down-regulated. Reactive oxygen species (ROS) was elevated significantly by formaldehyde in addition to markedly augmented membrane permeability of oocytes. These data suggest that formaldehyde contributes to edematous acute lung injury by reducing transalveolar Na^+^ transport, through decreased ENaC activity and enhanced membrane depolarization, and by elevating ROS production over long-term exposure.

Cigarette smoke is a complex mixture of more than 4,700 chemical compounds^1^,^2^,^3^ and contains carcinogens, free radicals, and highly toxic compounds that induce various lung impairments, such as apoptosis, oxidative stress, inflammation, and oncogenesis after respiratory inhalation^4^,^5^,^6^,^7^. Cigarette smoke causes acute lung epithelial injury^8^, characterized by pulmonary edema and chronic injury, including emphysema, bronchitis, and fibrosis^9^. Although chronic obstructive pulmonary disease patients generally do not exhibit typical clinical signs of lung edema, animal studies indicate that cigarette smoke exposure predisposes lungs to inflammation and acute lung injury^10^,^11^. Previous studies showed that cigarette smoke is a risk factor for the development of acute lung injury and lung edema may be another health consequence of cigarette smoke^12^. Of specific interest to our current study, the aldehydes in cigarette smoke may contribute to the development of edematous acute lung injury, chronic obstructive pulmonary disease, bronchitis, and fibrosis^4^,^7^,^13^.

In injured lungs, pulmonary edema occurs subsequent to an imbalance of alveolar fluid formation and fluid backflow to veins. Excess fluid accumulates in the air spaces of lung tissue and limits gas exchange across the blood-gas barrier, resulting in systemic severe hypoxia. It is likely that multiple insults that lead to dysfunctional alveolar salt and transalveolar fluid transport are involved in the pathogenesis of pulmonary edema^14^. Increasing evidence shows that epithelial sodium channels (ENaC), which are inhibited by the drug amiloride, play an important role in alveolar fluid clearance and edema fluid resolution^15^,^16^,^17^. Generally, the α-subunit forms electrically detectable Na^+^ channels, whereas the β- and γ-subunits are auxiliary subunits that increase the amplitude of the activity of the α-subunit channels by two orders of magnitude. Hummler *et al*. found that mice deficient in α-ENaC died shortly after birth with fluid-filled lungs^18^, and humans with certain genetic variants of ENaC are prone to developing pulmonary edema at high altitude^19^.

Among the aldehydes in cigarette smoke, formaldehyde is one of the most important; it is also a well-known, and common, indoor and outdoor pollutant. Exposure to formaldehyde has been linked to asthma, especially in children^20^, and formaldehyde can strengthen the asthmatic response to allergens in humans^21^. Concentration-dependent reductions in short-circuit current were induced by formaldehyde in excised canine and human bronchial epithelium tissues^22^. However, the direct effects of formaldehyde on lung ENaC have not been studied. In the present study, we tested the hypothesis that formaldehyde depresses ENaC as a mechanism of aldehyde-induced pulmonary edema, using H441 cells, oocytes and mice as our models for examining ENaC activity.

## Results

### Formaldehyde decreases mouse alveolar fluid clearance *in vivo*

**Figure 1:**
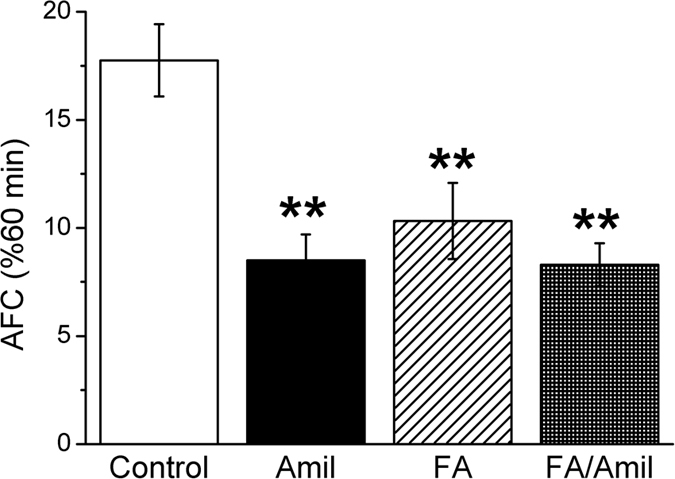
Downregulation of mouse alveolar fluid clearance *in vivo* by formaldehyde. Mouse lungs were instilled with 5% bovine serum albumin dissolved in physiologic saline solution in control group (Control), and in the presence of amiloride (Amil, 1 mM), formaldehyde (FA, 200 μM), and both (FA/Amil) for exposed mice. Reabsorption rate of instillate was computed as the percentage of instilled volume after 60 min (% 60 min). Average AFC values are presented as mean ± SE, One-way ANOVA. **P < 0.01, compared with control group. N = 6.

To investigate whether formaldehyde could inhibit alveolar fluid transport in the mouse lungs to develop lung edema, AFC was measured by instilling 5% BSA saline. As shown in Fig. 1, the *in vivo* AFC rate was reduced to 10.3% in the presence of formaldehyde, significantly lesser than controls (17.8 ± 1.7%, N = 6, *P *< 0.01). We next studied whether formaldehyde inhibits ENaC activity. To address this issue, we applied a specific ENaC inhibitor, amiloride (1 mM). Our data showed that amiloride reduced AFC to 8.5 ± 1.2% (*P *< 0.01 versus Control, N = 6). Coadministration of formaldehyde (FA/Amil) did not further reduce fluid resolution (8.3 ± 1.0%, *P *> 0.05 versus formaldehyde group, N = 6). These *in vivo* data suggest that formaldehyde could decrease amiloride-sensitive fraction of AFC associated with ENaC and formaldehyde might decrease transalveolar fluid clearance contributed by ENaC.

### Effects of formaldehyde on amiloride-sensitive short-circuit currents in intact H441 monolayers

**Figure 2:**
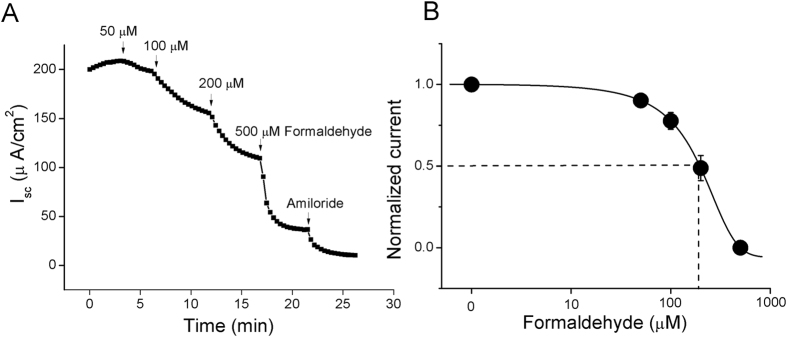
Formaldehyde reduces short-circuit current level in H441 monolayers in a dose-dependent manner. (**A**) Representative short-circuit current (I_sc_) trace showing applications of a series of concentrations of 50, 100, 200, and 500 μM formaldehyde. Amiloride-sensitive currents are defined as the difference between the total current and the amiloride-resistant current, with the basal amiloride-sensitive current set as 100%. (**B**) Normalized amiloride-sensitive currents at each concentration were plotted as a dose-response curve, N = 7. The raw data were calculated by fitting with the Boltzmann equation. The IC_50_ value was 165.6 μM.

Amiloride-sensitive component of AFC reflecting ENaC-mediated fluid transport and ENaC has been reported to contribute to ~60% of AFC^23^,^24^. We postulated that ENaC was a target of formaldehyde-induced lung edema. To examine the effects of formaldehyde on electrogenic transepithelial Na^+^ transport, we measured the amiloride-sensitive short-circuit currents in confluent H441 monolayers, which were mounted in an 8-chamber Ussing chamber system. H441 cells are derived from human Clara cells found in the bronchiolar epithelium. This cell line has been applied to study lung ENaC in last two decades^25^. Compared with primary cells, there are not between-donor variances since lung ENaC are regulated by sex hormones, aging, pollutants, and other noxious insults. Although H441 are cancerous cells, the airway epithelial cells in smokers are neoplasm. When formaldehyde was pipetted to the apical side of the monolayers, short-circuit current levels were inhibited in a dose-dependent manner, and the current was markedly inhibited by 100 μM amiloride, a specific inhibitor of sodium channels (Fig. 2A). We set the basal amiloride-sensitive current as 100%, with amiloride-sensitive currents defined as the difference between the total current and the amiloride-resistant current. Following treatment of H441 monolayers with 50, 100, 200, or 500 μM formaldehyde, the amiloride-sensitive short-circuit currents decreased to 97.9% (±0.6%), 91.1% (±2.3%), 78.2% (±6.2%), and 46.4% (±9.6%), respectively. The IC_50_ value was 165.6 μM (±0.5 μM), calculated by fitting the dose-response curve with the Boltzmann equation^26^ (Fig. 2B). These findings suggested that formaldehyde treatment decreased ENaC activity.

### Formaldehyde inhibits expression of the ENaC α-subunit protein in H441 cells

**Figure 3:**
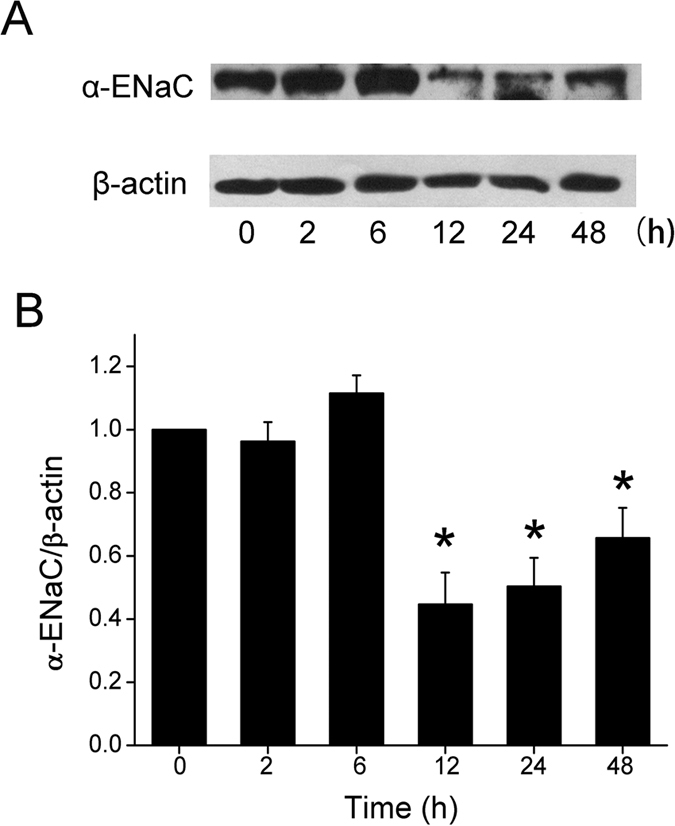
Effect of formaldehyde on α-ENaC protein level in H441 cells. (**A**) Representative western blot of α-ENaC protein extracted from H441 cells exposed to 200 μM formaldehyde for 0-48 h. Blots were immunostained with antibody to α-ENaC, or to β-actin as a loading control. (**B**) Graphical representation of data obtained from three sets of western blots and quantified using gray analysis (α-ENaC/β-actin). Data are shown as the mean ± SE, *P < 0.05, compared with control.

Per the genetic engineered mice, ‘scnn1a’, encoding ENaC α subunit, is critical for removing fluid from the air spaces. To determine whether formaldehyde exposure altered expression of the ENaC α-subunit at the protein level, we treated H441 cells with 200 μM formaldehyde for different periods. As shown by immunoblotting analysis (Fig. 3A), formaldehyde exposure decreased α-ENaC protein levels, whereas expression of the internal standard β-actin was unchanged. The specific band about 97 kD for the α-ENaC protein could be seen according to the manufacturer’s manual and the full-length blots/gels are presented in Supplementary Figure 1. Using β-actin levels as reference, α-ENaC protein expression was significantly reduced by 12 h, and although levels had risen by 48 h, they remained significantly lower than controls (Fig. 3B). We also examined γ-ENaC protein expression, but although a slight decrease was detected in treated cells, the levels were not significantly different from control levels (data not shown). We were unable to examine β-ENaC protein expression in cell lysates, due to the lack of an anti–β ENaC antibody suitable for western blot method.

### Formaldehyde inhibits mRNA expression of ENaC α- and γ-subunits in H441 cells

**Figure 4:**
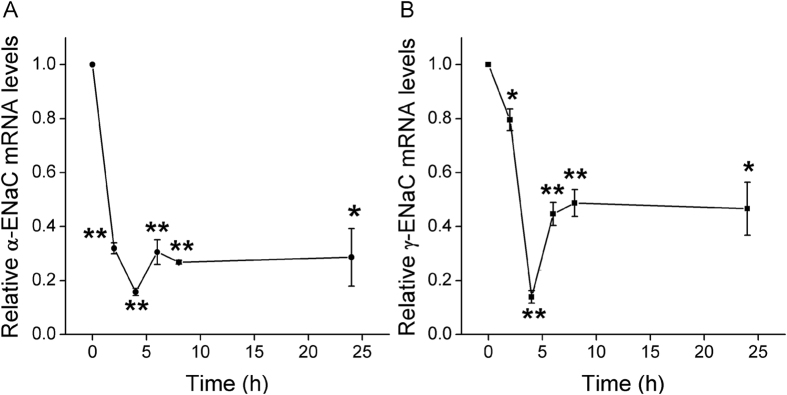
Effects of formaldehyde on transcriptional expression of ENaC α- and γ-subunits in H441 cells. mRNA samples were isolated from H441 cells treated with 200 μM formaldehyde for various periods. Relative levels of mRNA were calculated as α- or γ-ENaC/GAPDH ratios. (**A**) Real-time PCR results for α-ENaC mRNA. (**B**) Real-time PCR results for γ-ENaC mRNA. *P < 0.05, **P < 0.01, compared with control. N = 3.

Based on the reduction of ENaC protein level, we predicted a decrease in ENaC subunit at the transcriptional level. To examine whether formaldehyde inhibits ENaC expression in human alveolar epithelial cells at the transcriptional level, H441 cells were treated with 200 μM formaldehyde for different periods, and then α-ENaC and γ-ENaC expression was evaluated by real-time PCR. Figure 4A shows that formaldehyde inhibited α-ENaC mRNA expression rapidly at 2 h. By 4 h, α-ENaC mRNA levels had decreased to 15.7% of control cell levels. γ-ENaC mRNA expression was also suppressed quickly, falling to 13.9% of control cell levels by 4 h (Fig. 4B). These data demonstrate that formaldehyde inhibits α-ENaC and γ-ENaC mRNA expression in H441 cells in a time-dependent manner.

### Formaldehyde increases protein phosphorylation

**Figure 5:**
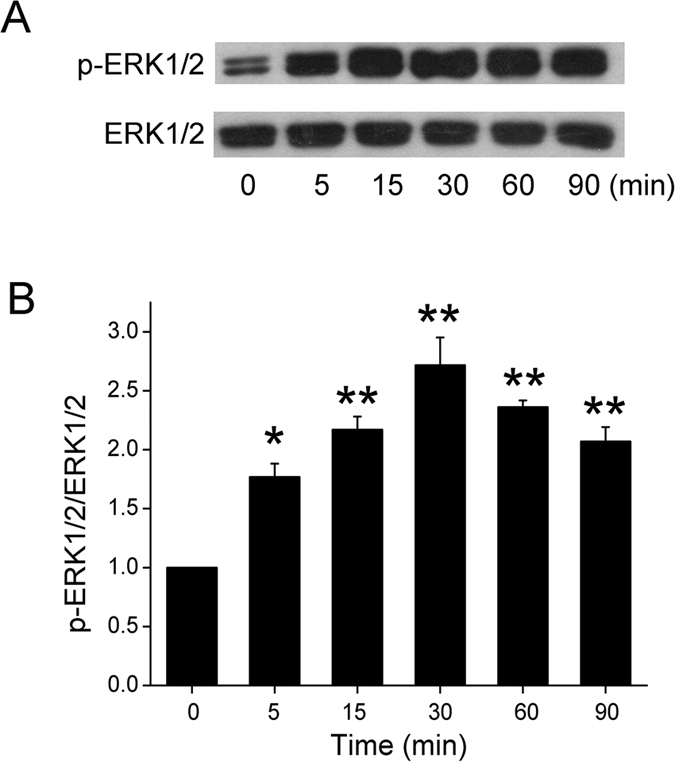
Effects of formaldehyde on ERK1/2 phosphorylation in H441 cells. (**A**) Representative western blot of phosphorylated ERK1/2 in protein extracted from H441 cells exposed to 200 μM formaldehyde for 0–90 min. Blots were immunostained with total ERK1/2 as a loading control. (**B**) Graphical representation of data obtained from three sets of western blots and quantified using gray analysis (p-ERK1/2/ERK1/2). Data are shown as the mean ± SE, *P < 0.05, **P < 0.01, compared with control.

**Figure 6:**
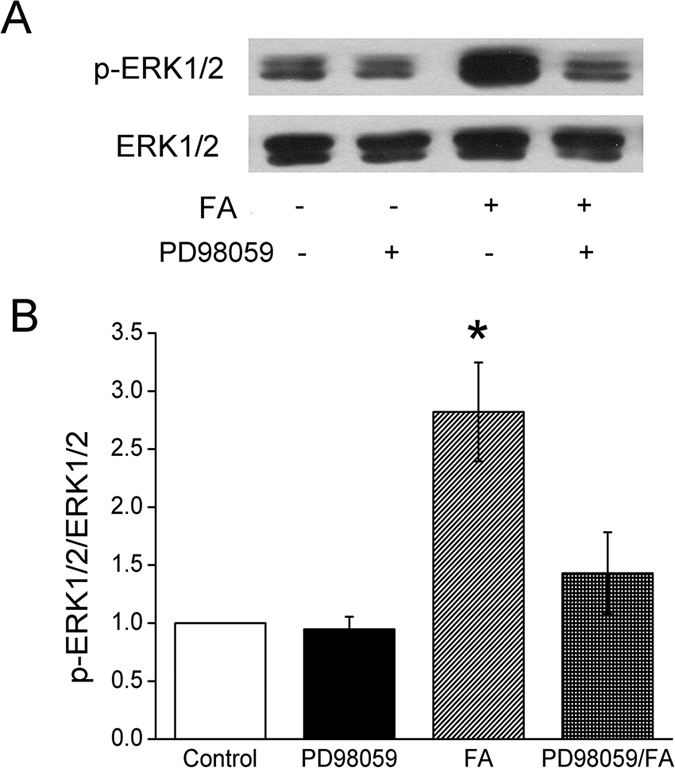
Effects of PD98059 on ERK1/2 phosphorylation in H441 cells. (**A**) Representative western blot of phosphorylated ERK1/2 in untreated H441 cells (Control), after pretreatment with PD98059 (PD98059), after treatment with formaldehyde (FA), and after pretreatment with PD98059 for 30 min prior to the addition of formaldehyde (PD98059/FA). Lanes shown in this figure are from the same western blot. (**B**) Graphical representation of data obtained from three sets of western blots and quantified using gray analysis (p-ERK1/2/ERK1/2). Data are shown as the mean ± SE, *P < 0.05, compared with control.

Phosphorylation of ENaC is the most common post-translational modification by noxious insults. We tested the possibility for formaldehyde to alter the balance of phosphorylation and dephosphorylation. The time-dependent effect of 200 μM formaldehyde on the phosphorylation of the extracellular regulated protein kinases 1 and 2 (ERK1/2) was then examined, which plays a key role in signal transduction and associates with ENaC activity. As demonstrated in Fig. 5A, formaldehyde elicited a rapid and strong induction of ERK1/2 phosphorylation at 5 min. Compared to total ERK1/2, phospho-ERK1/2 proteins reached maximal levels at 30 min (Fig. 5B, *P *< 0.01, N = 3). The full-length blots/gels are presented in Supplementary Figure 2. Treatment of cells with the ERK1/2 inhibitor PD98059 (100 μM) for 30 min before formaldehyde exposure decreased formaldehyde-enhanced ERK1/2 phosphorylation, suggesting that formaldehyde could reduce ENaC activity (Fig. 2A) by enhancing the phosphorylation of ERK1/2 (Fig. 6A,B).

### Formaldehyde increases oxidative stress in H441 cells

**Figure 7:**
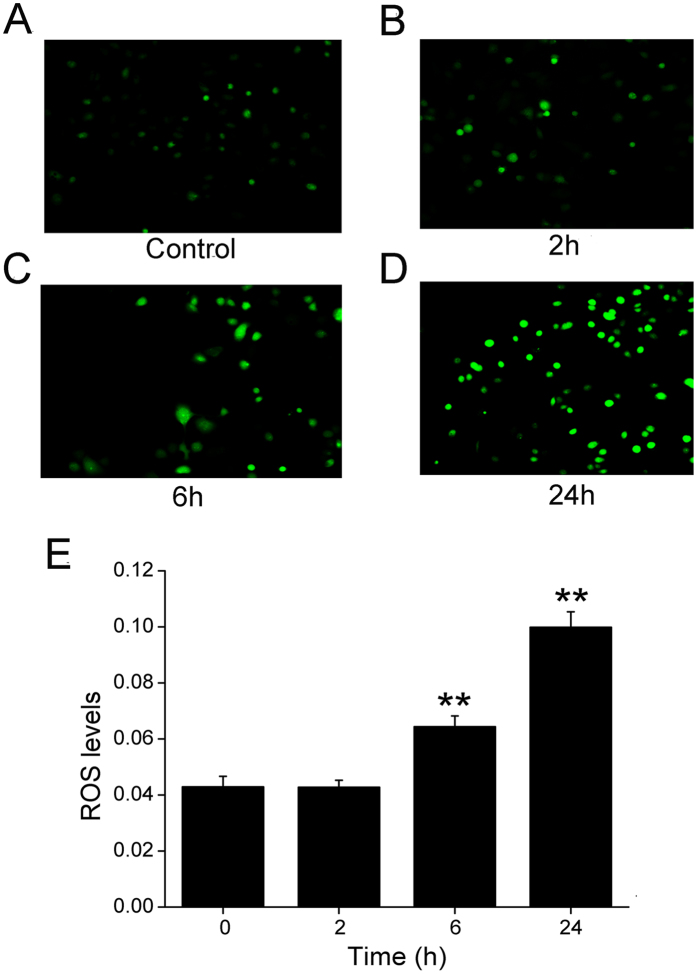
Effects of formaldehyde on oxidative stress in the H441 cells. Production of ROS was measured using the fluorogenic substrate 2′,7′-dichlorofluorescein diacetate, which is oxidized to fluorescent 2′,7′-dichlorofluorescein. (**A**) Air-subjected control cell culture. (**B**–**D**) Cells exposed to 200 μM formaldehyde for 2 h, 6 h, and 24 h. (**E**) The ROS levels displays the summary fluorescence intensity measured from the images of H441 cells (N = 25 in each group) treated with different periods of formaldehyde. Background fluorescence level was corrected. Data are shown as the mean ± SE, **P < 0.01, compared with control.

Cigarette smoke increases oxidative stress levels in the lung^27^, and based on that study, we speculated that potent oxidative formaldehyde, a component of cigarette smoke, might increase oxidative stress in H441 cells. Therefore, H441 cells were treated with 200 μM formaldehyde for 0–24 h, and intracellular oxidative levels were examined using the dichlorofluorescein assay. Figure 7 demonstrated that cells exposed to formaldehyde exhibited a time-dependent increase in ROS levels (A-D), with significant differences detected at 6 and 24 h post exposure compared to the unexposed controls (Fig. 7E).

### Formaldehyde inhibits human αβγ-ENaC activity in oocytes

**Figure 8:**
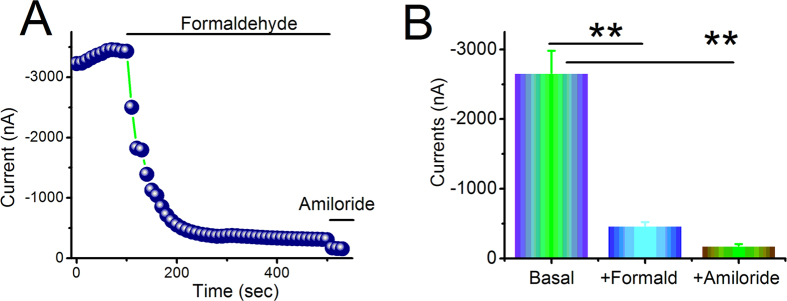
Formaldehyde alters the activity of heterologous human αβγ-ENaC channels expressed in oocytes. Oocytes were continuously perfused with formaldehyde and amiloride, and whole cell currents were monitored at 10-s intervals. (**A**) Representative inward current traces in the presence of formaldehyde and amiloride. Currents were digitized at −120 mV. Application of drugs is indicated by solid horizontal lines. (**B**) Average currents at −120 mV (mean ± SE). **P < 0.01 compared with basal level. N = 10 from 4 different frogs.

To examine the effects of formaldehyde on human αβγ-ENaC, oocytes expressing αβγ-ENaC were superfused with 200 μM formaldehyde. The inward and whole cell currents were decreased by formaldehyde and reached a stable plateau within a few minutes (Fig. 8A). The inhibited current at −120 mV was approximately 15% of the basal level, a statistically significant change (Fig. 8B, *P *< 0.01). In the presence of amiloride (10 μM), αβγ-ENaC activity was almost inhibited completely. These results suggest that formaldehyde can rapidly inhibit human αβγ-ENaC activity. We speculated that the rapid decrease of oocyte current and the short circuit current is associated with the specific inhibition of formaldehyde on ENaC-associated function, for only αβγ-ENaC channel is expressed in occytes and the amiloride-sensitive short circuit currents which mainly reflect ENaC activity in H441 monolayers are affected. Whereas the slow effect of formaldehyde on the oxidative stress shows the overall trend of all channels in H441 cells, including non-ENaC components.

### Formaldehyde depolarizes cells by increasing membrane permeability

**Figure 9:**
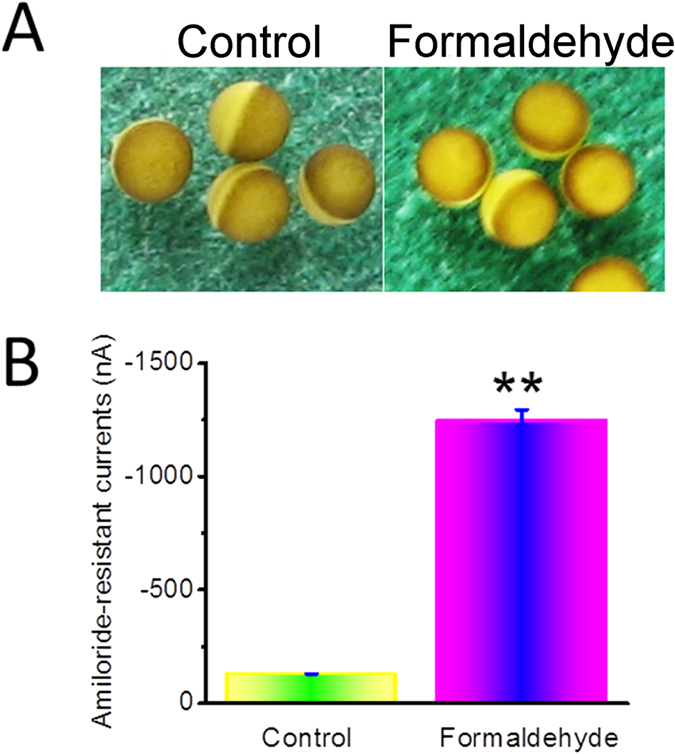
Formaldehyde impairs properties of the plasma membrane. Oocytes expressing human αβγ-ENaC were incubated with formaldehyde for 24 h. (**A**) The shape of oocytes expressing human αβγ-ENaC observed by microscopy, with and without formaldehyde exposure. (**B**) Average amiloride-resistant currents at −120 mV (mean ± SE), reflecting oocyte permeability. **P < 0.01 compared with control. N = 10 from 4 different frogs.

Incubation in formaldehyde for 24 h had little effect on the shape of oocytes expressing human αβγ-ENaC (Fig. 9A). However, analysis of amiloride-resistant currents revealed a significant increase in the currents in formaldehyde-pretreated oocytes compared to the control group (Fig. 9B). These data indicate that formaldehyde leads to an augmentation in membrane permeability of oocytes, but without causing visible structural damage.

## Discussion

Alveolar fluid clearance appears to play an important role in lung fluid balance in both physiological and pathological conditions, as evidenced by the study of Matthay’s group, which showed that patients with acute lung injury and acute respiratory distress syndrome (ALI/ARDS) have dysfunctional alveolar fluid clearance^28^. The increased permeability of capillary endothelium, resulting from injury to the lung microvascular circulation, may lead to increased transvascular fluid filtration into the perimicrovascular interstitium^14^. If the epithelial layer is also damaged, the fluid will move though the epithelial layer into the airspace^29^. While cystic fibrosis transmembrane conductance regulator deficiency had been shown to contribute to the physiopathology of cigarette-induced diseases such as chronic bronchitis and COPD^30^,^31^, most studies confirm that Na^+^ reabsorption is also a key factor in maintaining fluid balance in the lung, and ENaC plays a crucial role in Na^+^ reabsorption^32^,^33^,^34^.

The human lung adenocarcinoma cell line NCI-H441 is one of the classic cell lines used in researching ENaC activity and function. In the present study, H441 cells were used to examine the effects of formaldehyde, a component of cigarette smoke, on ENaC function. We found that formaldehyde could inhibit the amiloride-sensitive short-circuit current levels of H441 cell monolayers in a dose-dependent manner, suggesting that formaldehyde may inhibit Na^+^ transport by suppressing lung ENaC. We chose 200 μM formaldehyde in the following experiments according to the IC_50_ value calculated in our ussing chamber results and the previous studies^35^, which amounts to the tobacco smoke for 3~4 cigarettes^36^. We also studied the effects of formaldehyde on mouse alveolar fluid clearance, with results suggesting that formaldehyde reduced alveolar fluid clearance in mice.

Due to the importance of ENaC in lung fluid clearance and the major functional role of its α-subunit^18^, we examined whether formaldehyde had any effect on α-ENaC. Using H441 cells, we discovered that formaldehyde exposure decreased α-ENaC expression at the protein and transcriptional levels, and that γ-ENaC mRNA expression was also inhibited. Furthermore, we explored the influence of formaldehyde on protein phosphorylation of the ERK pathway. ERK protein, consisting of ERK1 and ERK2, is a member of the MAPK family, which plays a key role in signal transduction from surface receptors to the nucleus^37^. Previous studies demonstrated that the activation of ERK1/2 inhibited ENaC activity by phosphorylating residues in the C-termini of the β andγsubunits, enhancing the docking of the ubiquitin ligase (Nedd4-2) with these subunits^38^,^39^,^40^. It has also been reported that transforming growth factor-β1 reduced α-ENaC mRNA expression on apical membranes through the ERK1/2 pathway^41^. Additionally, formaldehyde induces apoptosis of lung epithelial cells through the p38MAPK pathway^42^. In this study, the phosphorylation of ERK1/2 was enhanced rapidly and strongly by exposure to formaldehyde, whereas treatment with the ERK1/2 inhibitor PD98059 inhibited this formaldehyde-induced phosphorylation of ERK1/2, suggesting that formaldehyde reduces ENaC activity via enhancing ERK1/2 phosphorylation in H441 cells.

Previous studies suggested the relevance of the ROS in the apoptosis induced by cigarette smoke extract and ROS production as a common mechanism of ENaC regulation by insulin^27^,^43^. Studies also have shown that cigarette smoke extract regulates lung ENaC via oxidant signaling, affecting alveolar fluid balance^44^. Because formaldehyde is one of the substances in cigarette smoke that can induce oxidative stress in the lung, we examined the role of formaldehyde on oxidative stress, using a fluorescent marker of oxidation. The results showed an increase in fluorescence in cells exposed to formaldehyde, suggesting that formaldehyde can increase intracellular oxidation levels. The apical membrane ENaC expression is known to be regulated by aldosterone and the amiloride-sensitive short-circuit currents mainly reflects ENaC activity, is also a function of the ion selectivity and permeability properties of the paracellular and transcellular pathways^45^,^46^. As we know, oocytes have little intrinsic channels and *Xenopus laevis* oocyte expression system has been widely used to elucidate the electrophysiological features of different kinds of channels by injecting the corresponding cRNAs. The biophysical properties and pharmacological profile of ENaC expressed in oocytes are similar to those of the native channel^47^. Another advantage of the oocyte expression system is to eliminate other native ion transport pathways, enable us to test the direct effects of formaldehyde on human lung ENaC. Our voltage clamp results using oocytes expressing αβγ-ENaC showed that formaldehyde exposure markedly augmented the membrane permeability of oocytes without apparent damage.

## Conclusions

These data suggest that formaldehyde sharply reduces ENaC activity via enhancing ERK1/2 phosphorylation and membrane depolarization, thus reducing transalveolar Na^+^ transport, followed by decreasing the transcription and translation of ENaC subunits, as well as elevating cellular ROS products over long-term exposure.

## Materials and Methods

### Animals

All experiment methods involving mice and *Xenopus laevis* were carried out in accordance with the guidelines and regulations of Animal Care and Use Committee and all experimental protocols were approved by China Medical University and the University of Texas Health Science Center at Tyler, respectively. Animals were kept under pathogen-free conditions. Please see following relevant paragraphs for experimental details.

### Air-Liquid Cell Culture

The human club cell line NCI-H441 was cultured as described previously^48^,^49^, in a humidified atmosphere of 5% CO_2_ and 95% O_2_ at 37 °C. RPMI-1640 medium (ATCC, Manassas, VA) was supplemented with 10% fetal bovine serum (Gibco, Waltham, MA), 2 mM L-glutamine, 10 mM HEPES, 1 mM sodium pyruvate, 4.5 g/l glucose, 1.5 g/l sodium bicarbonate, 100 IU/ml penicillin and 100 μg/ml streptomycin, and the culture medium was changed every other day. Cells were maintained in serum-free medium overnight after reaching 90% confluency, and then the medium was changed before exposure of the cells to formaldehyde. To analyze protein phosphorylation, cells were pretreated with 100 μM of the mitogen-activated protein kinase (MAPK) inhibitor PD98059 (Beyotime, Shanghai) for 30 min prior to formaldehyde exposure. For Ussing chamber assays, cells were grown in Costar Snapwell culture cups, until reaching confluency at 24 h, and then medium and non-adherent cells in the apical compartment were removed to adapt the cells to air-liquid interface culture. An epithelial volt/ohm meter (WPI, Sarasota, FL) was used to measure transepithelial resistance, and confluent filters with resistance >500 Ωcm^2^ were used for measuring short-circuit current levels.

### Treatment of H441 with Formaldehyde

Cultures of H441 cells were grown to 80–90% confluency. For formaldehyde time-dependent experiments, cells were treated at a final concentration of 200 μM for periods ranging from 0–48 h; this concentration of formaldehyde was chosen based on our Ussing chamber results and a previous report^50^. To detect intracellular ROS levels, cells were incubated for 2, 6, and 24 h at 37 °C with 200 μM formaldehyde, and ROS levels were analyzed according to the manufacturer’s instructions from the Reactive Oxygen Species Assay Kit (Beyotime, China).

### Ussing Chamber Assay

Measurements of transepithelial resistance and short-circuit currents in H441 monolayers were performed as described previously^51^. H441 monolayers were mounted in Ussing chambers (Physiologic Instruments, San Diego, CA) and bathed on both sides with a solution of 120 mM NaCl, 25 mM NaHCO_3_, 3.3 mM KH_2_PO_4_, 0.83 mM K_2_HPO_4_, 1.2 mM CaCl_2_, 1.2 mM MgCl_2_, and 10 mM HEPES, supplemented with either 10 mM mannitol (apical compartment) or 10 mM glucose (basolateral compartment)^24^. The osmolality of each solution, as measured by a freezing depression, was between 290 and 300 mOsm/kg. Both sides of the bath solutions (pH 7.4) were bubbled with 95% O_2_ and 5% CO_2_ continuously at 37 °C. The short-circuit current levels were measured with 3 M KCl, connected on either side to Ag-AgCl electrodes filled with 4% agarose salt bridges. H441 monolayers were short-circuited to 0 mV, and a 10 mV pulse of 1 s was given every 10 s to monitor transepithelial resistance. Data were collected using the Acquire and Analyse program version 2.3.

### Western Blot Analysis

Cells were pretreated with formaldehyde and then lysed, and extracted proteins were electrophoresed on 8% polyacrylamide gels before transferring to PVDF membranes (Invitrogen, Waltham, MA). After a 1-h incubation in a blocking solution containing 20 mM Tris-Cl (pH 7.5), 0.5 M NaCl, and 5% BSA, membranes were incubated with primary antibodies: α-ENaC antibody (Thermo Fisher, Cat. no. PA1-920A); antibodies against phospho-ERK1/2 (Cat. no. 4370) and total ERK1/2 (Cat. no. 4695) (Cell Signaling Technology, Danvers, MA); or β-actin antibody (Santa Cruz Biotechnology, Cat. no. sc-47778). Primary and secondary antibodies were diluted 1:1000 and 1:2000, respectively, and membranes were incubated with antibodies in a buffer containing 5% BSA, 20 mM Tris-Cl (pH 7.5), 0.5 M NaCl, and 0.1% Tween 20, at 4 °C overnight. Horseradish peroxidase-conjugated secondary antibodies were incubated at room temperature for 1 h, and three 10-min washes in TBST [20 mM Tris-Cl (pH 7.5), 0.5 M NaCl, 0.1% Tween 20] were applied after each antibody reaction. Images were developed using an enhanced chemiluminescence (ECL) kit, and data were collected using the Image J program.

### Real-time PCR

For real-time PCR, H441 cells were seeded in RPMI-1640 medium containing 10% FBS, in 6-well plates, and grown to about 80–90% confluence. After exposure to formaldehyde for different time periods, cells were washed with cold PBS. Total RNA was extracted using TRIzol reagent (Invitrogen, Waltham, MA), according to the manufacturer’s instructions, and RNA concentration was measured by spectrophotometry at 260 nm. The following primer pairs were used for real-time PCR: α-ENaC forward (5′-AAC AAA TCG GAC TGC TTC TAC-3′) and α-ENaC reverse (5′-AGC CAC CAT CAT CCA TAA A-3′), γ-ENaC forward (5′-GCA CCG TTC GCC ACC TTC TA-3′) and γ-ENaC reverse (5′-AGG TCA CCA GCA GCT CCT CA-3′), and GAPDH forward (5′-AGA AGG CTG GGG CTC ATT TG-3′) and GAPDH reverse (5′-AGG GGC CAT CCA CAG TCT TC-3′). Total RNA (1 μg) was used as a template for each reaction, and all samples were run in triplicate in a final volume of 20 μl. Reverse transcription was performed using a single cycle of 95 °C for 15 min, followed by 40 cycles of 95 °C for 1 min, 55 °C for 0.5 min, 72 °C for 0.5 min. Relative expression of ENaC mRNA was calculated using the 2^−△(△CT)^ comparative method, with normalization of each sample against expression of the endogenous reference gene GAPDH.

### Detection of Intracellular ROS

The fluorogenic substrate 2′,7′-dichlorofluorescein diacetate is a cell-permeable dye, which, after entering into a cell, can be hydrolyzed into non-fluorescent 2′,7′-dichlorofluorescein by intracellular esterases and then oxidized to fluorescent 2′,7′- dichlorofluorescein by ROS within the cytosol; the resulting change in fluorescence intensity can be used to measure intracellular generation of ROS. To assay for ROS, H441 cells were cultured in 24-well plates, as described above. After exposure to 200 μM formaldehyde for 24 h, the cells were washed twice with PBS and then incubated in medium containing 10 μM 2′,7′-dichlorofluorescein diacetate for 30 min at 37 °C in the dark. Immediately after incubation, the cells were washed three times with serum-free medium to remove extracellular 2′,7′-dichlorofluorescein diacetate, and fluorescence intensity was monitored using a Leica DMI 3000B inverted fluorescence microscope.

### Expression of human ENaC in oocytes

Oocytes were surgically removed from appropriately anesthetized adult female *Xenopus laevis* (Xenopus Express, Brooksville, FL), and cRNAs for α, β, and γ human ENaC were prepared as described previously^52^. Briefly, frogs were placed under anesthesia by administering ethyl 3-aminobenzoate methanesulfonate salt (Sigma, Louis, MO) through a small incision in the lower abdomen, and then ovarian tissue was removed. Follicle cells were removed and digested in Oocyte Ringer solution 2 (OR-2) calcium-free medium (82.5 mM NaCl, 2.5 mM KCl, 1.0 mM MgCl_2_, 1.0 mM Na_2_HPO_4_, and 10.0 mM HEPES, pH 7.5) with the addition of 2 mg/ml collagenase (Roche, Indianapolis, IN). Defolliculated oocytes were cytosolically injected with ENaC cRNAs (25 ng per oocyte) in 50 nl of RNase-free water, at a subunit ratio of 1α:1β:1γ, and incubated in half-strength L-15 medium at 18 °C. Control cells were not injected with cRNA.

### Two-electrode voltage clamp assay

The two-electrode voltage clamp technique was used to record whole-cell currents 48 h after injection^53^. Oocytes were impaled with two electrodes filled with 3 M KCl and with resistances ranging from 0.5–2.0 MΩ; a TEV-200 voltage clamp amplifier (Dagan, Minneapolis, MN) was used to clamp the oocytes and record currents. Two reference electrodes were connected to the bath, which contained a bathing solution of ND96 medium (96.0 mM NaCl, 1.0 mM MgCl_2_, 1.8 mM CaCl_2_, 2.5 mM KCl, and 5.0 mM HEPES, pH 7.5) for continuous perfusion. Experiments were controlled using pCLAMP 10.1 software (Molecular Devices, Sunnyvale, CA), and currents of −40, −100, and +80 mV were continuously monitored at intervals of 10 seconds. The current traces were acquired by stepping the holding potential to −120 mV after the monitoring currents were stable. Data were sampled at the rate of 200 Hz and filtered at 500 Hz.

### Measurement of *in vivo* alveolar fluid clearance in mice

AFC was measured as previously described^54^. In brief, BALB/c male mice (20–30 g) were anaesthetized with diazepam (17.5 mg kg^−1^, intraperitoneally) followed 6 min later by ketamine (450 mg kg^−1^, intraperitoneally) and were placed on a heating pad. The trachea was exposed and cannulated with a trimmed 18-gauge intravenous catheter, which was then connected to a mouse respirator (HX-300, Chengdu Taimeng Technology Co. Ltd, Chengdu, China). Mice were ventilated with 100% O_2_ with a 200 μl tidal volume (8–10 ml kg^−1^) at a 160 breaths min^−1^. Once stable anaesthesia was obtained, mice were positioned in the left decubitus position, and 300 μl of isosmolar NaCl or 200 μM formaldehyde containing 5% fatty acid-free bovine serum albumin was instilled *via* the tracheal cannula, followed by 100 μl of room air to clear dead space. After instillation, mice were ventilated for a 60 min period, and then the alveolar fluid was aspirated. All reagents were added to the AFC instillate from stock solutions directly before instillation, in a minimal volume of solvent (1–10 μl ml^−1^). The protein concentrations in aspirated solutions were measured by Bradford Method. We estimated AFC (% AFC_60_) by the changes of concentration for bovine serum albumin as water was absorbed after 60 min. AFC was calculated as the follow equation: AFC = [(Vi − Vf)/Vi] × 100, where V is the volume of the instilled bovine serum albumin solution (i) and the final alveolar fluid (f). Vf = Vi × Pi/Pf, where P represents the protein concentration in the instilled bovine serum albumin solution (i) and the final alveolar fluid (f).

### Statistical analysis

Data were expressed as the mean ± SE. One-way ANOVA computations were used to analyze the difference of the means for normally distributed data. Mann-Whitney test was applied for nonparametric data. Significance levels were *P < 0.05 and **P < 0.01.

## Additional Information

**How to cite this article**: Cui, Y. *et al*. Formaldehyde impairs transepithelial sodium transport. *Sci. Rep.* **6**, 35857; doi: 10.1038/srep35857 (2016).

## Supplementary Material

Supplementary Information
