# Supplementary material for: Formaldehyde impairs transepithelial sodium transport
Source: Sci Rep. 2016 Oct 20;6:35857. doi: 10.1038/srep35857 (PMC5071906; doi:10.1038/srep35857)

Formaldehyde impairs transepithelial sodium transport

Yong Cui[1,#], Huiming Li[2,#], Sihui Wu[2], Runzhen Zhao[3], Deyi Du[2], Yan Ding[2], Hongguang Nie[2,*], Hong-Long Ji[3,*]

[1]Department of Anesthesiology, First Affiliated Hospital of China Medical University, Shenyang, Liaoning, China; [2]Institute of Metabolic Disease Research and Drug Development, China Medical University, Shenyang, Liaoning, China; [3]Department of Cellular and Molecular Biology, University of Texas Health Science Center at Tyler, Tyler, Texas, USA

Running title: Formaldehyde inhibits ENaC

#These authors contributed equally to this work.

*Corresponding Authors:

Dr. Hongguang Nie, PhD, MD. hgnie@cmu.edu.cn and Dr. Hong-Long Ji, MD, PhD. james.ji@uthct.edu.

Supplementary Figure 1. The full-length blots/gels of α-ENaC protein extracted from H441 cells exposed to 200 μM formaldehyde for 0-48 h. Blots were immunostained with antibody to α-ENaC, or to β-actin as a loading control.

Supplementary Figure 2. The full-length blots/gels of phosphorylated ERK1/2 in protein extracted from H441 cells exposed to 200 μM formaldehyde for 0-90 min. Blots were immunostained with total ERK1/2 as a loading control.

Supplementary Figure 1

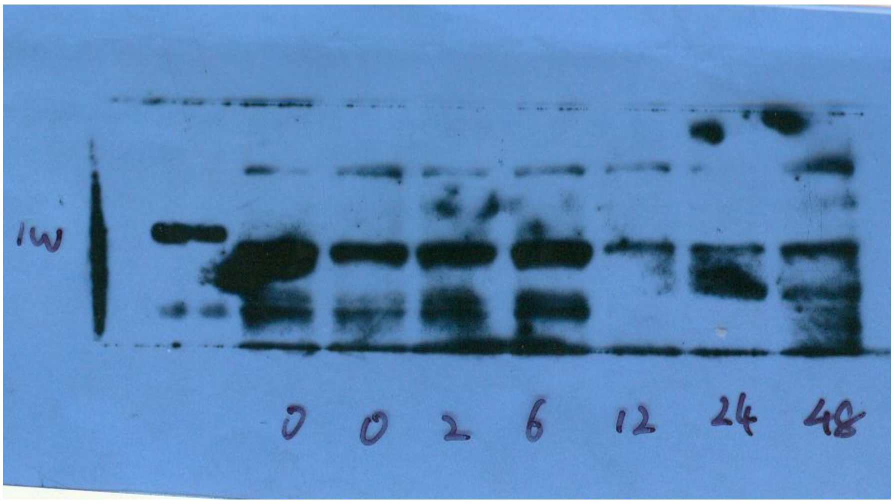

Supplementary Figure 2

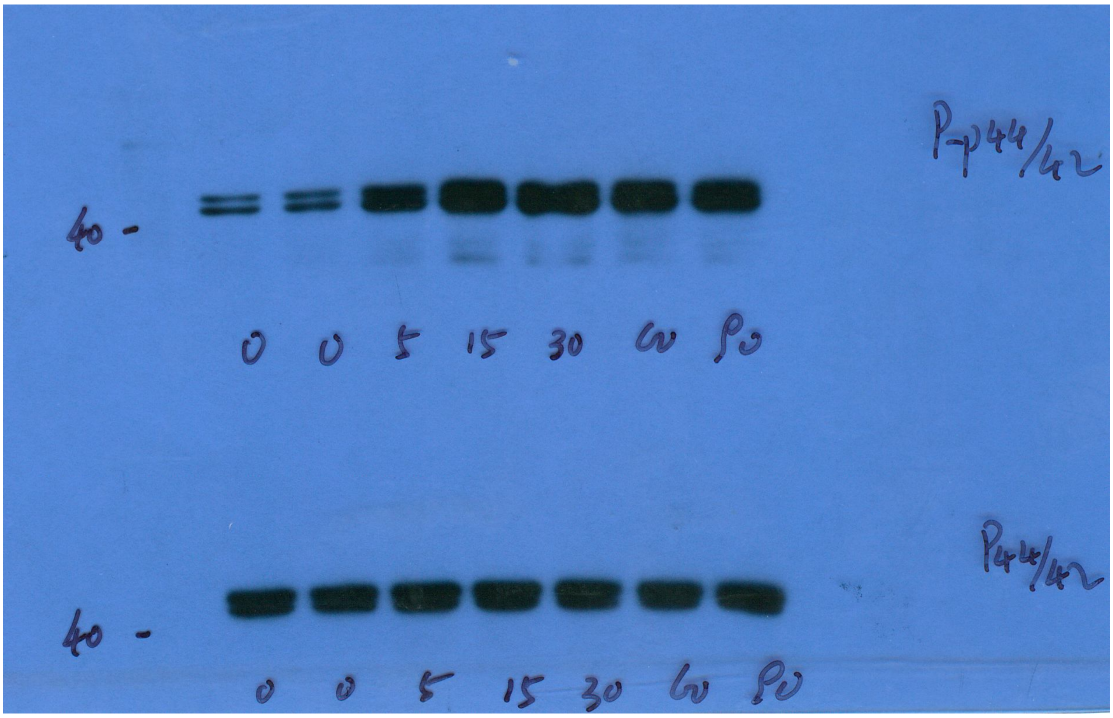

Supplement: Supplementary Information [file srep35857-s1.pdf]
